# Repeat gamma knife radiosurgery for recurrent trigeminal neuralgia: a systematic review and meta-analysis

**DOI:** 10.1007/s00701-026-06891-7

**Published:** 2026-05-01

**Authors:** Maria Fernanda P. Santana, Lucca B. Palavani, Yusuf-Zain Ansari, Natan Lucca Lima, Lara Lucena Fernandes Filgueiras, Isabela Montenegro Tenório de Carvalho, Larissa Karla Santos de Santana Alves, Gustavo Joji Yoshida, Ana Beatriz B. Cavenaghi, Wagner Rios-Garcia, Willian Alberto Amaro Marchioli, Danilo Silva

**Affiliations:** 1 University Center of Várzea Grande, Várzea Grande, Mato Grosso Brazil; 2 Max Planck University Center, Indaiatuba, São Paulo Brazil; 3 Temple University, Philadelphia, PA USA; 4 Federal University of Santa Catarina - UFSC, Araranguá, BR Brazil; 5 Nova Esperança Faculty of Medicine, João Pessoa, Paraíba Brazil; 6 Federal University of Alagoas, Maceió, Alagoas Brazil; 7 Federal Fluminense University, Niterói, Rio de Janeiro Brazil; 8 Faculty of Medicine, National University of San Luis Gonzaga, Ica, Peru; 9 Department of Neurosurgery, New Hyde Park, Northwell Health, NY USA

**Keywords:** Gamma Knife, Stereotactic radiosurgery, Trigeminal neuralgia

## Abstract

**Introduction:**

Trigeminal neuralgia is a disorder of the trigeminal nerve that results in intense episodic pain. Medical management is commonly used as the first therapeutic intervention; however, some patients become resistant to it, requiring further interventions. Stereotactic radiosurgery with the Gamma Knife (GK) is safe and effective in multiple retrospective series. However, in some cases, repeat radiosurgery may be needed, aiming to improve the patient’s pain in the long term.

**Methods:**

We performed a systematic review and meta-analysis following the PRISMA guidelines. We included papers in English that reported patients with trigeminal neuralgia who didn’t have their pain solved with standalone medications or a single dose of GK, needing repeat GK. All statistical analyses were conducted using R.

**Results:**

Twenty-one studies met the inclusion criteria, totaling 2,486 patients initially treated and 2,020 undergoing repeat GKRS. The majority were female, with median ages 54.5–79.6 years, and idiopathic TN represented ~ 85% of cases. Most procedures targeted the trigeminal root entry zone using 70–80 Gy marginal doses. Pain relief occurred in 70–90% of patients, with marked improvement in BNI scores (typically IV–V to I–IIIb). Median follow-up ranged 14–74 months, and recurrence occurred in 10–35% of cases. Facial sensory disturbances were reported in 20–45%, while bothersome dysesthesias and anesthesia dolorosa were rare (< 3%). Meta-regression, including up to 10 studies, found no significant correlation between pain relief and age (*p* = 0.5682), pain duration (*p* = 0.3593), interval since last GKRS (*p* = 0.4093), or dose (*p* = 0.7857). Heterogeneity remained high (I^2^ = 55.6–91.8%, *p* < 0.0001), indicating inter-study variability.

**Conclusion:**

Repeat GKRS is a safe and effective option for TN patients experiencing persistent or recurrent pain after initial radiosurgery. Despite inter-study variability, outcomes support its use for long-term pain control. Further prospective studies are needed to optimize patient selection and treatment parameters.

**Supplementary Information:**

The online version contains supplementary material available at 10.1007/s00701-026-06891-7.

## Introduction

In the International Classification of Headache Disorders, third edition, trigeminal neuralgia is described as recurring, brief, electric shock–like pain on one side of the face, starting and ending suddenly, confined to one or more trigeminal nerve branches, and triggered by harmless stimuli [10]. Accurate identification of TN is the foundation for effective management [10, 22]. For medically refractory TN, Gamma Knife radiosurgery (GKRS) is now considered a first-line option [17, 18, 29, 30]. As a minimally invasive procedure, it carries a lower risk of complications than other surgical approaches and achieves favourable pain control (BNI I–IIIb) in over 75% of patients at short-term follow-up [17, 29, 34, 39]. However, recurrence rates exceeding 20% have been consistently reported, underscoring the need to recognize potential treatment failure and to evaluate alternative strategies, including repeat GKRS [1, 18, 35].

Current evidence supports this technique as an effective and minimally invasive treatment for medically refractory trigeminal neuralgia TN, achieving good pain control after both initial and repeat procedures [1, 2, 8, 11, 18, 35]. Positive response and mild facial numbness after the first GKRS are recognized predictors of successful retreatment [1, 11, 18]. However, recurrence remains frequent, and cumulative radiation doses and proximal targeting raise the risk of trigeminal nerve dysfunction [1, 2, 18, 33]. The long-term safety and efficacy of multiple GKRS sessions-especially beyond the second-are still uncertain due to small, heterogeneous studies [1, 21, 33]. Optimal dose reduction, target positioning, and thresholds that balance efficacy with sensory preservation remain debated, and prognostic factors for outcomes after multiple treatments have yet to be consistently validated [1, 18, 23, 33].

Nevertheless, the present study aimed to conduct a systematic review and meta-analysis assessing the effectiveness of Repeat Gamma Knife Radiosurgery for Trigeminal Neuralgia.

## Methods

This systematic review and meta-analysis were performed following the Cochrane Handbook for Systematic Reviews of Interventions and the Preferred Reporting Items for Systematic Reviews and Meta-Analysis (PRISMA) statement guidelines [14, 26]. It was registered at the International Prospective Register of Systematic Reviews (PROSPERO), ID:.

### Eligibility criteria

This systematic review and meta-analysis included all studies reporting the repeated use of the Gamma Knife for trigeminal neuralgia, with a minimum long-term follow-up of 12 months, as defined by the studies' screening criteria. Additionally, non-English papers, letters, comments, reviews, and meta-analyses were excluded.

### Search strategy and data extraction

A systematic search was conducted of the Medline, PubMed, Scopus, and Web of Science using the following terms: “repeat,” “recurrent,” “salvage,” “radiosurgery,” “stereotactic radiosurgery,” “Gamma Knife,” “gamma-knife,” “GKRS,” “trigeminal”. The data extraction process was independently conducted by three authors (L.L, Y.A., and L.K.), adhering to predefined search criteria (Table S1).

### Endpoints

Outcomes included primary endpoints, defined as clinical outcomes directly related to treatment efficacy, and secondary outcomes, which are safety and additional treatment-related outcomes. The primary outcomes analysed were: mean post-repeat GKRS pain numeric rating scale scores [24], the Barrow Neurological Institute (BNI) pain intensity score [31], after prior treatment, after GKRS, and at the latest follow-up; pain recurrence, indicating how many patients experienced the recurrence of the pain after the repeat GK intervention; and follow-up mean. Secondary outcomes were defined as complications associated with GK procedures; adverse effects, and the mean of the repeat GK dose, important for dose–response analysis. Good pain scale mean was defined as a reduction of ≥ 50% in pain intensity from baseline, considering the numerical scale of 0–10, being zero: no pain; and 10: worst pain possible. Additionally, for the BNI score, the good outcome was defined as BNI I and II.

### Statistical analysis

All statistical analyses were performed using RStudio software (version 4.2.3; R Foundation for Statistical Computing, Vienna, Austria). A single-arm meta-analysis was conducted using odds ratios (ORs) with 95% confidence intervals (CIs), and a *p* value < 0.05 was considered statistically significant. Meta-analyses were performed using a generalized linear mixed-effects model (GLMM), which is recommended for pooling proportions and single-arm outcomes. Because GLMMs estimate study effects using maximum likelihood methods rather than inverse-variance weighting, individual study weights are not presented in the forest plots.

Statistical heterogeneity among studies was assessed using the I^2^ statistic, with values < 25% indicating low heterogeneity. For outcomes demonstrating substantial heterogeneity, sensitivity analyses were conducted by sequentially excluding one study at a time to evaluate the stability of the pooled estimates.

To explore potential sources of heterogeneity, meta-regression analyses were performed using study-level covariates, including sample size, mean age, follow-up duration, and intervention dose, to determine whether these variables significantly influenced the outcomes. Publication bias was assessed through visual inspection of funnel plots and Egger’s test for outcomes with at least 10 studies included.

### Quality assessment

Two authors (M.F.P.S. and W.R.) independently evaluated the study quality, and any differences in their assessment were resolved through consensus. ROBINS-I, as recommended by the Cochrane Collaboration, evaluates bias across seven domains: bias due to confounding, selection of participants, classification of interventions, deviations from intended interventions, missing data, measurement of outcomes, and selection of the reported result. Each study was judged as having low, moderate, serious, or critical risk of bias, or no information, according to the tool’s guidance.

## Results

### Study selection

**Fig. 1:**
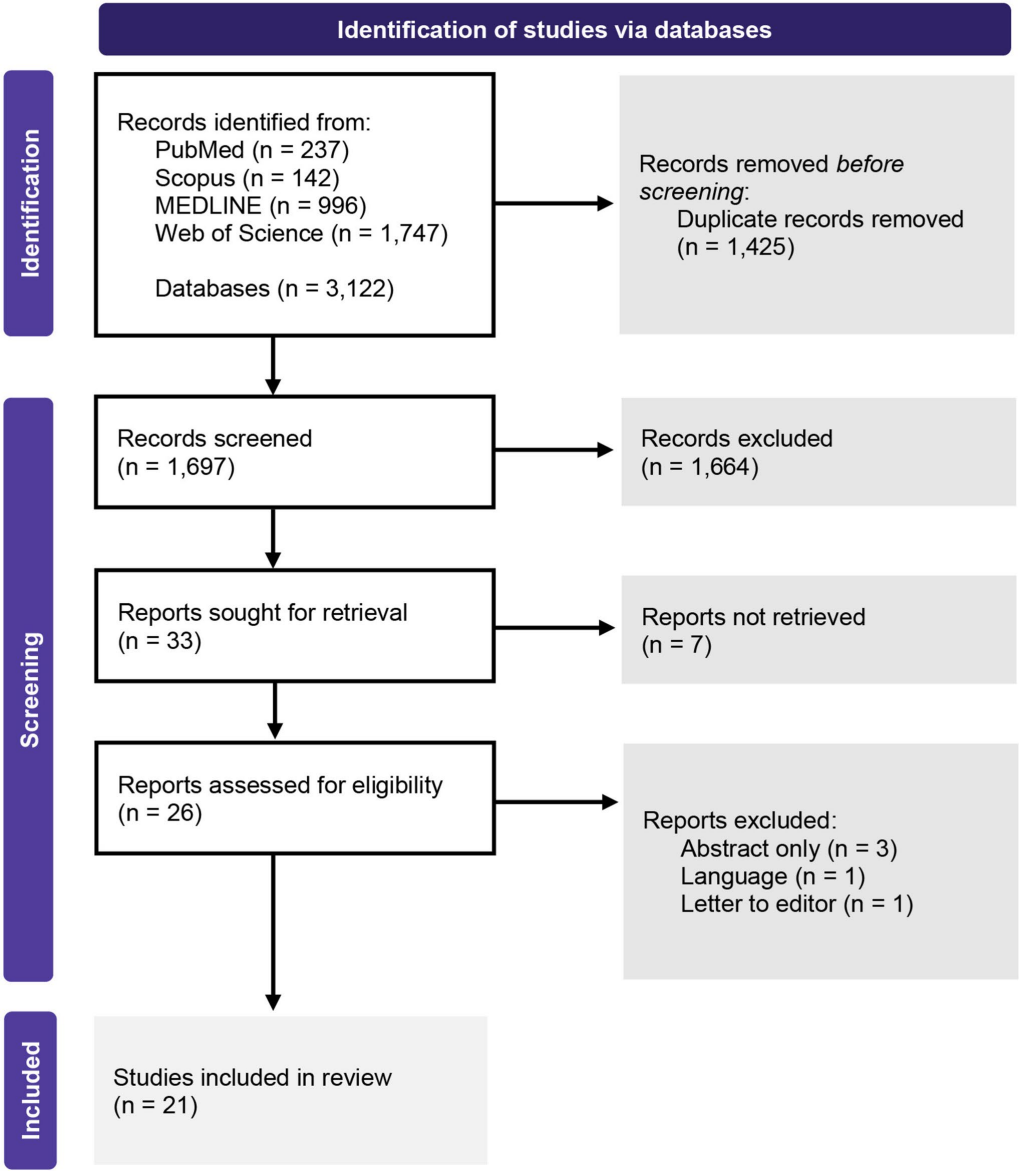
PRISMA flowchart of study selection

A comprehensive search across multiple databases identified 3,122 records, including 996 from MEDLINE, 237 from PubMed, 142 from Scopus, and 1,747 from Web of Science. After removal of duplicate records, 1,697 unique studies remained for screening. During title and abstract screening, 1,664 articles were excluded because they did not meet the inclusion criteria, including studies that were not related to Gamma Knife radiosurgery for trigeminal neuralgia, did not evaluate repeat GKRS procedures, were non-clinical studies, or were reviews, case reports, or conference abstracts without primary outcome data. The remaining 33 articles were assessed for full-text eligibility. Of these, 7 studies could not be retrieved despite attempts to obtain the full text. The remaining 26 full-text articles were evaluated in detail, and 5 studies were excluded for various reasons. Ultimately, 21 studies met the inclusion criteria and were included in the final analysis. Among the included studies, 3 were cohort studies and 18 were retrospective studies. The study selection process is illustrated in Fig. 1 (PRISMA flow diagram).

### Risk of bias results

**Fig. 2:**
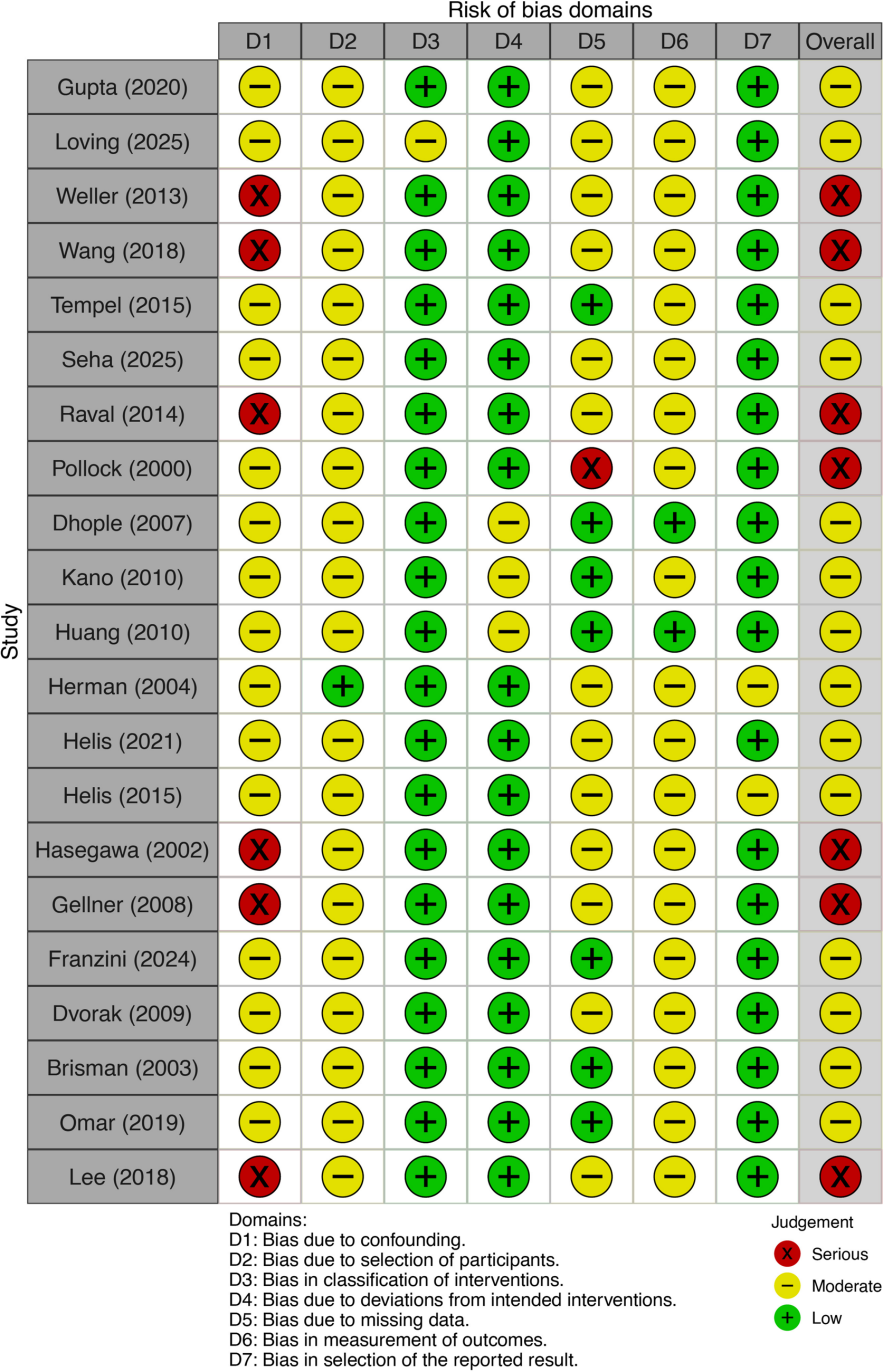
Traffic-Light plot for risk of bias assessment using the ROBINS-I tool

The risk of bias, assessed using the ROBINS-I tool in 21 studies, revealed a clear distribution. In the overall assessment, 14 studies were rated as having a "Moderate" risk, while 7 studies were classified as having an overall "Severe" risk of bias. When analyzing the domains, bias due to confounding factors (D1) was the main determinant of the overall "Severe" ratings, as 6 of the 7 studies with a severe overall risk were also rated as "Severe" in D1. The other domains showed consistently low levels of concern for D3 (Classification of Interventions), D4 (Intervention Biases), and D7 (Outcome Measurement), which were almost universally rated as "Low." Domains D2 (Participant Selection), D5 (Missing Data), and D6 (Outcome Measurement) were predominantly rated as "Moderate" in most studies, as shown in Fig. 2.

### Baseline characteristics of included studies

The analysis comprised a total of 2,486 patients who initially underwent Gamma Knife radiosurgery (GKRS) and 2,020 patients who underwent GKRS again for recurrent trigeminal neuralgia (TN). Among these patients, 1,240 were female and 662 were male, although gender distribution was not reported in every study. Regarding the patient’s age, in studies with records, the range spanned from 54,5 to 79,6 years, with an overall mean 67,5 years and with the most reporting median ages in the sixth decade of life. Approximately 85% of cases (*n* = 1,616.7) were idiopathic TN, while secondary etiologies, such as TN related to multiple sclerosis, were less frequent. Moreover, most repeat procedures targeted the trigeminal root entry zone (REZ), and the median marginal dose ranged from 70 to 80 Gy. About the procedures, the interval between them varied widely, from 5 to 138 months (median ≈ 36 months). Furthermore, the median follow-up varied from 14 to 74 months across the studies (Table 1).

**Table 1:** Baseline characteristics and patients' GKRS status

Author, year	Design	Study period	N of patients	M/F	Age at first GK	Age at repeat GK	Pain duration since prior GK (mo)	Time since last GK (mo)
Helis, 2015 [11]	Cohort	1999–2013	152	63/89	68	70	NA	15–16
Brisman, 2003 [2]	Retro	1998–2003	45	20/25	70 (40–90)	NA	117	18
Tempel, 2015 [33]	Retro	1995–2012	17	7/10	NA	79.6 (51–96)	NA	NA
Gupta, 2021 [7]	Retro	1997–2019	30	14/16	69 (42–86)	76.5 (47–94)	120	33 (5–138)
Loving, 2025 [20]	Retro	2012–2018	53	16/37	NA	70 (62–81)	NA	15 (9–29)
Lee, 2018 [19]	Retro	2006–2014	108	37/71	65 (22–90)	NA	60	NA
Weller, 2013 [38]	Retro	2002–2010	35	14/21	62 (39–86)	NA	36	NA
Wang, 2018 [37]	Retro	2008–2015	700	307/351	48.2 (31–76)	NA	NA	NA
Seha, 2025 [32]	Cohort	2015–2019	25	7/18	NA	68 (44–84)	75.6	NA
Pollock, 2002 [27]	Retro	1997–2002	29	NA	NA	NA	NA	NA
Raval, 2014 [28]	Cohort	2000–2006	354	NA	62	NA	NA	NA
Dhople, 2007 [3]	Retro	1996–2001	25	NA	55	60	NA	18.5
Kano, 2010 [16]	Retro	1992–2008	193	74/119	70 (26–93)	NA	120	45
Huang, 2010 [15]	Retro	1999–2008	65	31/34	61 (34–84)	NA	39	25
Herman, 2004 [13]	Retro	1996–2001	18	6/12	62	NA	51	8
Helis, 2021 [12]	Retro	2010–2018	22	10/12	NA	74.7	NA	48
Hasegawa, 2002 [9]	Retro	1993–2000	27	8/19	68.7	NA	124.8	22.3
Gellner, 2008 [6]	Retro	1994–2006	21	NA	NA	NA	NA	18.8
Franzini, 2024 [5]	Retro	2014–2022	18	6/12	54.5 (49–61)	58.5 (53–65)	60	22.5
Dvorak, 2009 [4]	Retro	2003–2006	28	14/14	NA	63	NA	18.1
Omar, 2019 [25]	Retro	1996–2012	55	23/32	NA	65.4	123.4	25.7

*F* Female, *GK* Gamma Knife, *M* Male, *mo* months, *N* Number of patients, *NA* Not available; Retro: Retrospective study

### Pain relief and functional outcomes

**Fig. 3:**
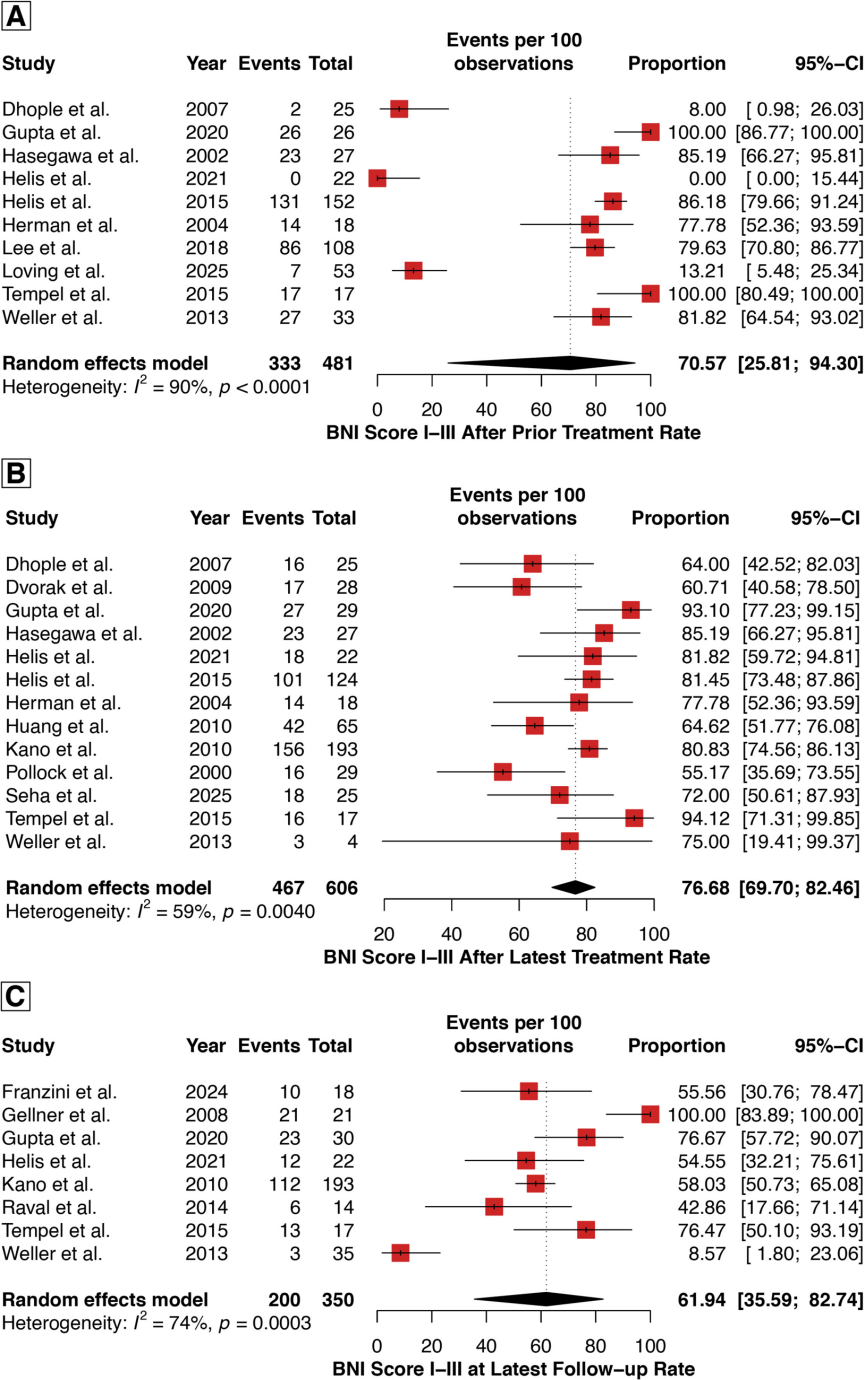
Forest plots for the analyses of (**A**) BNI Score I–III after prior treatment, **B** BNI Score I–III after latest treatment, and (**C**) BNI Score I–III after latest follow-up

The pooled proportion of patients achieving favourable pain outcomes (BNI I–III) after prior treatment is shown in Fig. 3A, with a rate of 70.57% (95% CI 25.81–94.30) and substantial heterogeneity (I^2^ = 90%, *p* < 0.0001). Leave-one-out analysis identified Loving et al. as the study contributing most to heterogeneity (Figure S1B). Assessment of publication bias using the funnel plot (Figure S7A) and Egger’s test (*p* = 0.4604705) did not indicate significant publication bias.

Following the latest treatment, the pooled proportion of favourable outcomes (BNI I–III) increased to 76.68% (95% CI 69.70–82.46; Fig. 3B), with moderate heterogeneity (I^2^ = 59%, *p* = 0.004). Leave-one-out analysis did not identify any individual study exerting a predominant influence on heterogeneity (Figure S2B). Publication bias assessment through the funnel plot (Figure S7B) and Egger’s test (*p* = 0.8298978) showed no evidence of asymmetry.

At the last available follow-up, the pooled rate of favourable pain control (BNI I–III) decreased to 61.94% (95% CI 35.59–82.74; Fig. 3C), with high heterogeneity (I^2^ = 74%, *p* = 0.0003), suggesting partial loss of treatment effect over time. Leave-one-out analysis identified Weller et al. as the main contributor to heterogeneity (Figure S3B).

### Pain relief and recurrence

**Fig. 4:**
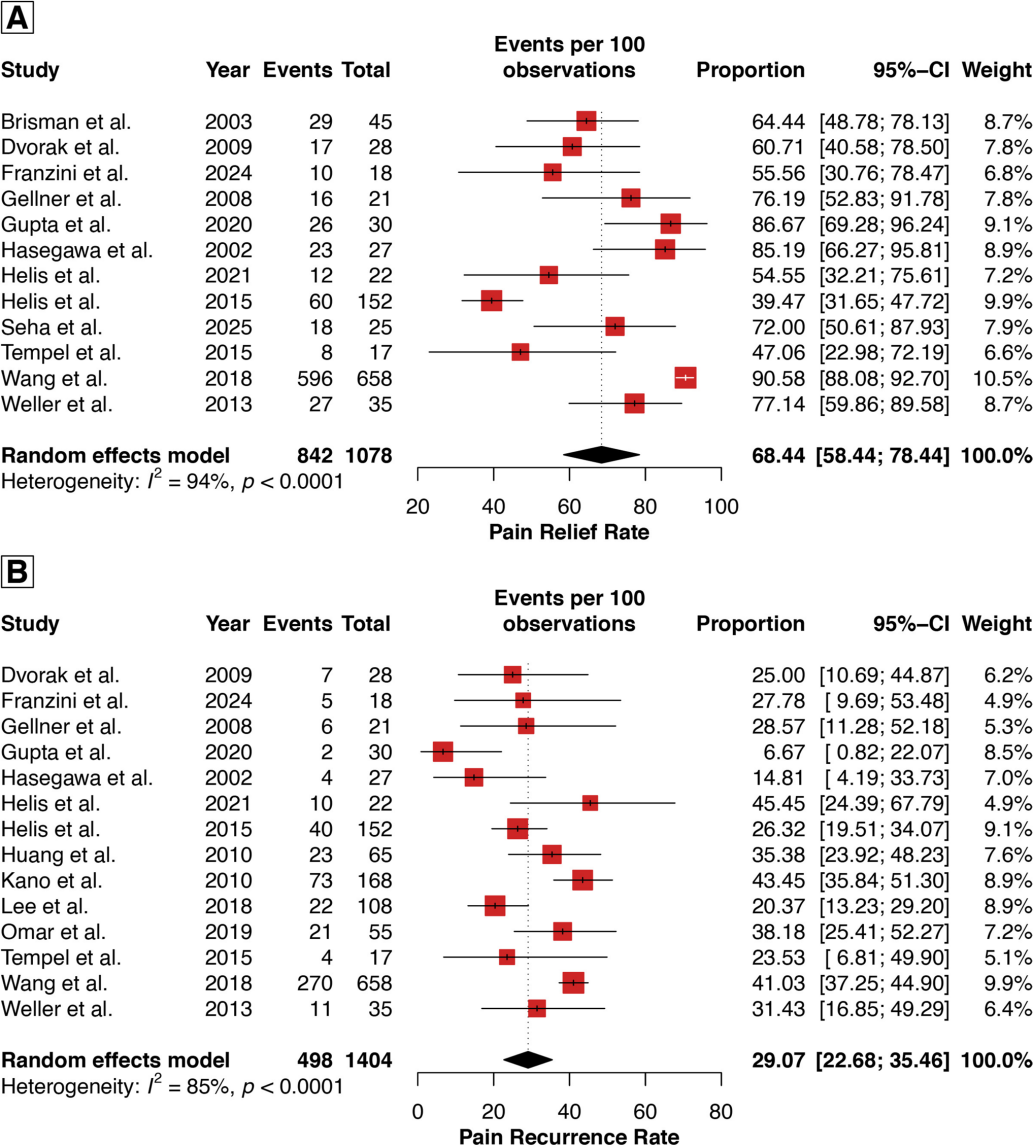
Forest plots for the analyses of (**A**) Pain relief, and (**B**) Pain recurrence

Pain relief outcomes are presented in Fig. 4A, with a rate of 68.44% (95% CI 58.44–78.44) and substantial heterogeneity (I^2^ = 94%, *p* < 0.0001). Leave-one-out analysis identified Hellis et al. (2015) as the study contributing most to heterogeneity (Figure S4B). Meta-regression analyses evaluating potential moderators — including age at first treatment, age at last treatment, pain duration, time since last treatment, and radiation dose — did not identify any statistically significant associations with pain relief (Table S2). Funnel plot inspection (Figure S7C) suggested asymmetry, and Egger’s test indicated possible publication bias (*p* = 0.027443).

Pain recurrence after repeat GKRS is summarized in Fig. 4B, with a rate of 29.07% (95% CI 22.68–35.46) and substantial heterogeneity (I^2^ = 85%, *p* < 0.0001). Leave-one-out analysis identified Gupta et al. as the study contributing most to heterogeneity (Figure S5B). Meta-regression analyses of the same covariates (age at first treatment, age at last treatment, pain duration, time since last treatment, and radiation dose) again showed no statistically significant associations with recurrence risk (Table S3). Funnel plot assessment (Figure S7D) and Egger’s test (*p* = 0.1743075) suggested possible publication bias.

### Adverse events

**Fig. 5:**
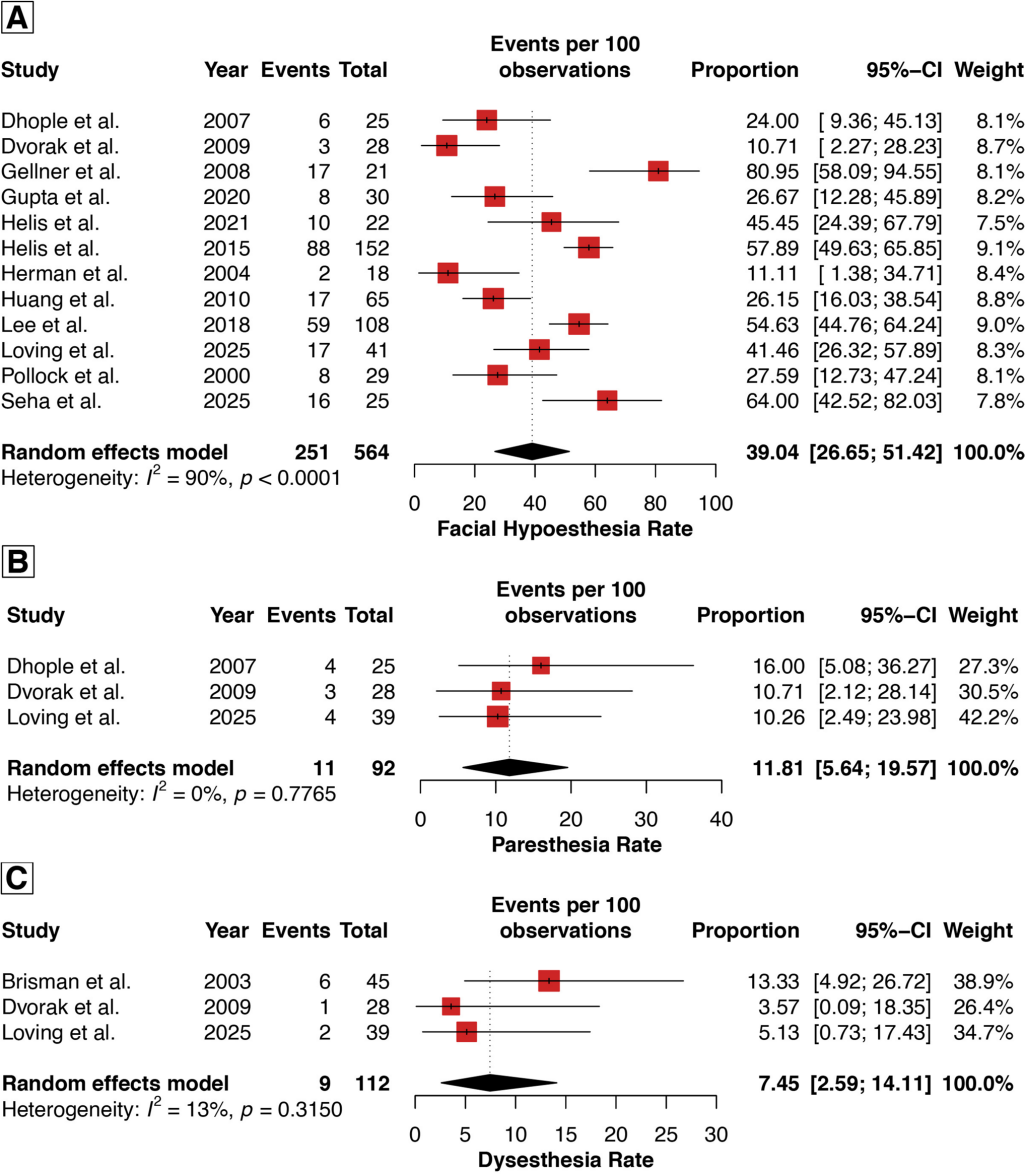
Forest plots for the analyses of (**A**) Facial hypoesthesia, **B** Paresthesia, and (**C**) Dysesthesia

The most frequent complication was facial hypoesthesia, with a pooled rate of 39.04% (95% CI 26.65–51.42; I^2^ = 90%; Fig. 5A). Leave-one-out analysis did not identify any study exerting a predominant influence on heterogeneity (Figure S6B). Funnel plot inspection (Figure S7E) and Egger’s test (*p* = 0.536939) did not indicate significant publication bias. The pooled analysis of paresthesia was 11.81% (95% CI 5.64–19.57; I^2^ = 0%; Fig. 5B). Dysesthesia was also reported with a pooled rate of 7.45% (95% CI 2.59–14.11; I^2^ = 13%; Fig. 5C).

**Fig. 6:**
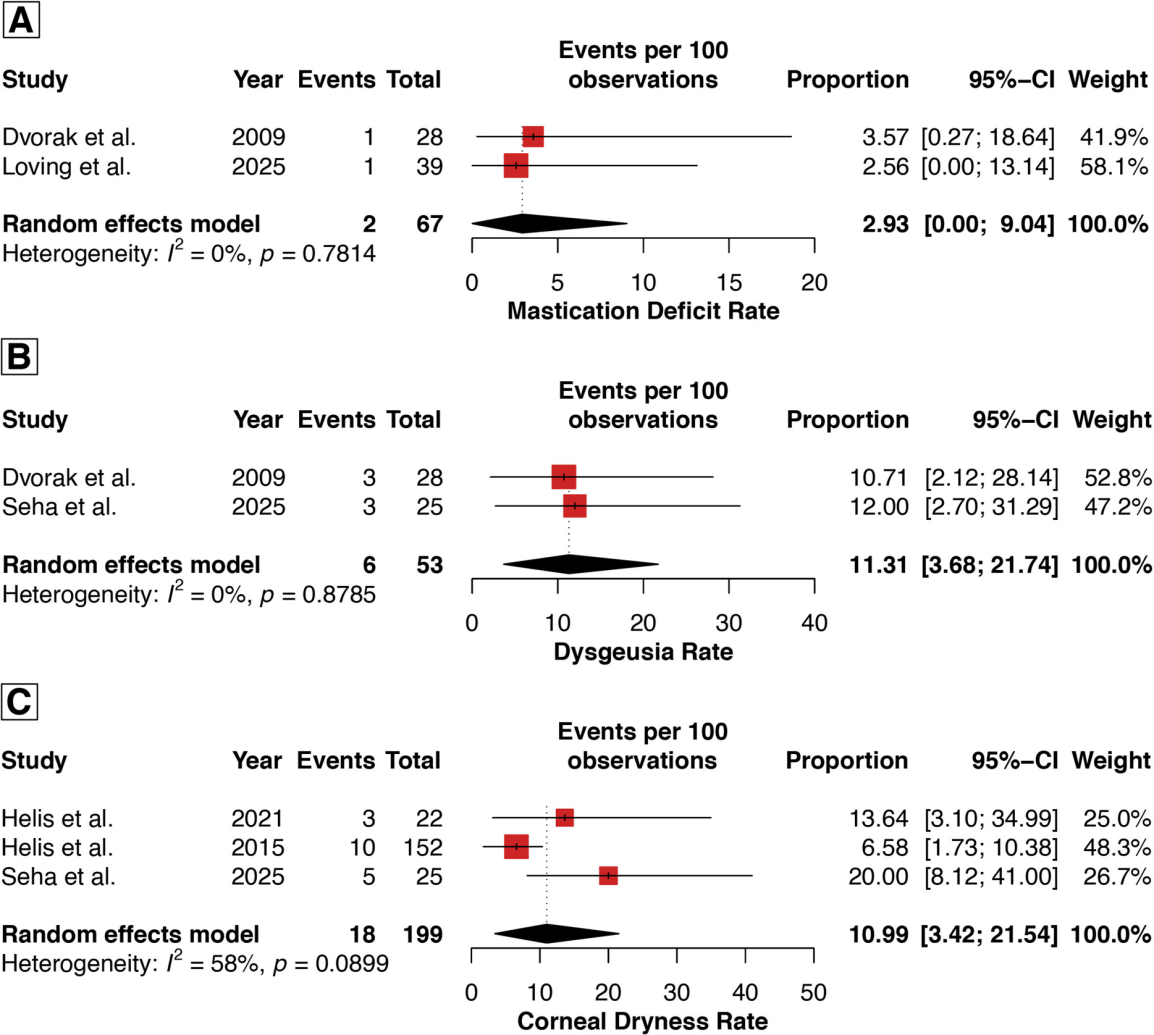
Forest plots for the analyses of (**A**) Masticatory deficit, **B** Dysgeusia, and (**C**) Corneal dryness

Additional complications included masticatory deficit (2.93%, 95% CI 0–9.04; I^2^ = 0%; Fig. 6A), dysgeusia (11.31%, 95% CI 3.68–21.74; I^2^ = 0%; Fig. 6B), and corneal dryness (10.99%, 95% CI 3.42–21.54; Fig. 6C). No deaths or permanent neurological deficits were reported across the included studies.

## Discussion

This meta-analysis consolidates current evidence on the outcomes of repeat Gamma Knife radiosurgery (GKRS) for trigeminal neuralgia (TN), demonstrating that a second radiosurgical procedure achieves favourable pain relief (BNI I-III) in 77% of patients, with sustained benefit in 62% at long-term follow-up. These results suggest that repeat GKRS may be a safe and effective minimally invasive option for patients experiencing pain recurrence after the initial procedure, particularly for those who may be unsuitable for open surgery.

Our pooled outcomes are consistent with prior reports on repeat GKRS. Helis et al. [11] described 84% pain control after retreatment, while Brisman et al. [2] observed relief in 62% of cases. The present meta-analysis found that repeat GKRS was associated with a pooled favourable pain control rate (BNI I–III) of 77%, suggesting that roughly three out of four patients may experience meaningful pain improvement following repeat treatment. However, long-term follow-up showed a modest decline in efficacy, with sustained pain relief observed in 62% of cases, consistent with gradual recurrence over time. These findings suggest that even after multiple GKRS sessions, meaningful pain reduction may still be achieved with a relatively low risk of new trigeminal sensory dysfunction, indicating that cumulative irradiation does not necessarily result in increased morbidity.

Regarding complications, facial hypoesthesia appeared to be the most frequently reported adverse effect following repeat Gamma Knife radiosurgery (39%), followed by paresthesia (12%), dysgeusia (11%), corneal dryness (11%), dysesthesia (7.5%), and masticatory deficit (3%). No deaths or permanent neurological deficits were reported across the included studies. These findings are consistent with prior large series, where sensory dysfunctions rates ranged from 20 to 65% after one or more procedures. In the multicentre analysis by Gupta et al. [7], new or progressive bothersome facial numbness occurred in 25% of patients after the first GKRS, 18% after the second GKRS, and 14% after the third GKRS treatment, and bothersome numbness was reported in 14% of patients after the third GKRS treatment. Similarly, after a second GKRS, Loving et al. [20] found that 61.5% of patients had chronic sensory deficits, primarily facial numbness (44%) and numbness complications related to paraesthesia (10%). When combined, these results place the meta-analysis’s pooled estimate within the range of significant published series, confirming that facial hypoesthesia is still the most frequent, but usually manageable, side effect of radiosurgical treatment.

These results, when looked at considering evidence, show that repeat GKRS remains a valuable treatment for patients with trigeminal neuralgia refractory to other modalities. In the included studies, most repeat procedures used marginal doses in the range of 70–80 Gy, reflecting current practice patterns aimed at balancing pain control with the risk of sensory toxicity. In line with Lee et al. [19], a dose range of 70–90 Gy targeted to the root entry zone achieved pain relief in about 90% of the patients, though new facial numbness occurred in 55%, highlighting the balance between efficacy and safety.

The lack of substantial predictors in our meta-regression (age, dose, pain, duration, or inter-procedure interval) suggests that treatment outcomes are primarily influenced by individual anatomical and radiobiological factors rather than demographic characteristics. Lee et al. [19] further showed that shorter pain history (≤ 5 years) predicted better pain relief and faster response, while other variables such as age and gender were not significant.

Therefore, accurate target selection and meticulous dose adjustment are crucial to maximize pain relief while reducing sensory morbidity, especially in patients who have undergone multiple GKRS sessions. The trigeminal root entry zone is the most common radiosurgical target, but even minor variations in its location or dose distribution can significantly influence both treatment efficacy and the risk of sensory complications. As cumulative radiation doses approach 160 Gy, the threshold commonly associated with an increased risk of facial hypoesthesia and dysesthesia, further re-irradiation demands careful planning to avoid overlapping high-dose regions. The consideration underscores the importance of individualized dosimetry, guided by prior treatment maps and precise image fusion, to ensure that the analgesic benefit on repeat GKRS is achieved without exceeding safe radiobiological limits.

A recent systematic review and meta-analysis by Valeri et al. [36] addressed the same clinical question and should be acknowledged alongside the present study [36]. Their review included 13 studies and 461 patients, focused primarily on outcomes after a second stereotactic radiosurgery (SRS), and reported BNI ≤ III in 73% of patients after repeat treatment, 31.9% relapse among responders, and 43.8% trigeminal sensory dysfunction. In contrast, our review included a larger and more contemporary evidence base (21 studies; 2,020 repeat GKRS procedures), incorporated newer series, and used a broader proportion-based synthesis with separate pooled analyses for pain relief after prior treatment, after repeat GKRS, at last follow-up, recurrence, and individual adverse events, in addition to leave-one-out sensitivity analyses and meta-regression. Additionally, Valeri et al. judged most studies as low-to-moderate risk with only a few studies at serious risk, whereas our ROBINS-I assessment was more conservative, with 14 studies rated moderate and 7 severe overall, largely driven by confounding and participant-selection concerns. These methodological differences likely explain some of the variation in presentation and interpretation; however, the overall message is consistent across both reviews: repeat GKRS can provide meaningful pain relief for recurrent or refractory TN, but interpretation is limited by retrospective study design, heterogeneous target and dose strategies, inconsistent reporting, and the need for more standardized future studies.

### Limitations

The strengths of the present study include a large, pooled cohort of 2,020 retreatments, strict inclusion criteria, and quantitative synthesis of both efficacy and adverse effects. The comprehensive statistical approach, including sensitivity analysis and meta-regression, provides an overview of treatment patterns and heterogeneity sources.

Despite its strengths, this meta-analysis has inherent limitations that warrant careful interpretation of the results. Although the pooled analysis provides a quantitative synthesis of the available evidences, several analyses demonstrated moderate statistical heterogeneity (I^2^ < 25%), suggesting minor methodological variability among the included studies which may influence the interpretation of pooled estimates. Differences in dosimetry parameters, target localization within the root entry zone, imaging guidance, and follow-up duration likely contribute to between-study variation.

In addition, most studies were retrospective in design and demonstrated moderate to severe risk of bias, which limited control over potential confounders and prevented uniform data collection across centres. Pain outcomes were consistently assessed using the Barrow Neurological Institute (BNI) Pain Intensity Scale, which, although widely adopted, remains a subjective tool that may introduce variability in interpretation among studies.

Another important limitation relates to clinical heterogeneity in patient populations. Patients with both idiopathic and multiple sclerosis trigeminal neuralgia were included. Because MS-related TN has a distinct pathophysiology and may respond differently to radiosurgical treatment, pooling these populations may introduce bias in the overall estimates. However, the vast majority of included cases were idiopathic trigeminal neuralgia with multiple sclerosis-related cases being less frequent, meaning these factors may be unlikely to have significantly influenced overall outcomes. Future studies should aim to report outcomes separately for these biologically distinct entities to allow more precise comparisons.

Similarly, variability existed in the number of repeat radiosurgical procedures included across studies. While most analyses focused on second GKRS procedures, some cohorts included patients undergoing third or subsequent GKRS treatments, which may have different efficacy and complication profiles due to cumulative radiation exposure and prior interventions. The inconsistent reporting of outcomes stratified by the number of repeat procedures limited our ability to perform subgroup analyses based on the number of radiosurgical treatments.

Additionally, meta-regression models included only 6–10 studies each, which limits statistical power and restricts definitive conclusions regarding potential predictors such as age, radiation dose, pain duration, or interval between procedures.

Given these limitations, the pooled results should be interpreted as an overall synthesis of the available literature rather than definitive estimates of treatment efficacy. Future prospective studies with standardized reporting of treatment parameters and patient characteristics, including separate analyses of idiopathic and MS-related trigeminal neuralgia, are needed to better clarify the factors influencing outcomes after repeat Gamma Knife radiosurgery.

## Conclusion

This meta-analysis suggests that repeat GKRS may provide meaningful pain relief for many patients with TN experiencing persistent or recurrent pain after initial treatment, with an acceptable safety profile. However, substantial heterogeneity among studies and the predominance of retrospective evidence warrant cautious interpretation of these findings.

Future research should aim to refine the parameters that determine optimal outcomes after repeated GKRS for TN. Prospective and, ideally, multicenter studies are needed to validate dose–response profiles, establish neural dose constraints, and identify predictors of durable pain control. The standardization of outcome reporting, particularly regarding the use of the BNI Pain Intensity Scale and definitions of recurrence, will be essential to improve data comparability across centres. Furthermore, the incorporation of technological advancements and refined targeting strategies may substantially enhance treatment precision and minimize sensory complications. Finally, collaborative registries combining clinical, radiobiological and imaging data could enable predictive modelling and facilitate individualized treatment planning in the future (Table 2).

**Table 2:** Intervention and clinical outcomes

Author, year	GK target	GK repeat dose (Gy)	Pain relief after repeat GK	BNI after prior GK	BNI after repeat GK	Adverse effects	Pain recurrence after repeat GK	Follow-up (mo)
Helis, 2015 [11]	REZ	80–85	Majority BNI I–IIIb	I–V	I–V	Numbness common	40	27
Brisman, 2003 [2]	Cistern	40	29 pts	NA	NA	Dysesthesia 8–4%	NA	36
Tempel, 2015 [33]	REZ 3–4 mm	70 (40–80)	8 pts	I–IIIb	I–IV	Sensory 5 pts	4	23
Gupta, 2021 [7]	REZ	81 (65–85)	26 pts	I–III	I–V	Sensory + twitching	2	39
Loving, 2025 [20]	Cistern/RGZ	70	NA	III–V	NA	Facial sensory 43%	NA	18
Lee, 2018 [19]	REZ	NA	NA	I–IIIb	NA	Facial numbness 59	22	43
Weller, 2013 [38]	REZ	NA	27 pts	I–V	I–IV	Sensory 39%	11	39
Wang, 2018 [37]	REZ	NA	90.5% relief	NA	NA	12 Complications	20	74
Seha, 2025 [32]	REZ	70–80	18 pts	Marseille I–III	NA	Hypoesthesia 64%	NA	26
Pollock, 2002 [27]	REZ	46.6	NA	I–IIIb	NA	Numbness 42%	NA	13
Raval, 2014 [28]	Cistern	45	5/14 excellent	NA	NA	NA	NA	58
Dhople, 2007 [3]	REZ	70	84%	III–V	I–V	Eye/ear 20–24%	NA	53
Kano, 2010 [16]	REZ	60–90 (80 main)	78–81%	I–IIIb	IV–V	Sensory 9%	NA	36
Huang, 2010 [15]	REZ	49 (35–80)	65 pts	I–V	NA	Numbness 26%	23	64
Herman, 2004 [13]	REZ	70 (65–75)	NA	I–V	I–V	Numbness 11%	NA	37
Helis, 2021 [12]	REZ/Pons	75–80	12 pts	IV–V	I–V	Numbness 10	10	40
Hasegawa, 2002 [9]	REZ	64.4	23 pts	I–IV	I–IV	Sensory 3	4	20
Gellner, 2008 [6]	REZ	74.3	16 pts	NA	I–III	Numbness 17	6	64
Franzini, 2024 [5]	REZ	85 (75–80)	10 pts	NA	I–V	Sensory 7	5	36
Dvorak, 2009 [4]	Cistern	45 (40–50)	17 pts	NA	NA	Mild sensory 29%	7	20
Omar, 2019 [25]	REZ	NA	NA	Marseille I–V	NA	NA	21	14

*GK* Gamma Knife, *REZ* Trigeminal Nerve Root Entry Zone, *RGZ* Retro-Gasserian Zone

## Supplementary Information

Below is the link to the electronic supplementary material.

ESM 1 Supplementary Material 1 (PDF 1.05 MB)

## Data Availability

No datasets were generated or analysed during the current study.
