# Supplementary material for: Repeat gamma knife radiosurgery for recurrent trigeminal neuralgia: a systematic review and meta-analysis
Source: Acta Neurochir (Wien). 2026 May 1;168(1):143. doi: 10.1007/s00701-026-06891-7 (PMC13284032; doi:10.1007/s00701-026-06891-7)

Repeat Gamma Knife Radiosurgery for Recurrent Trigeminal Neuralgia: A Systematic Review and Meta-Analysis

Supplementary Material

Table of Contents:

Table S1: Search Strategy……………………………………………………………………………...

Figure S1: BNI Score After Prior Treatment Analysis……………………………………………………...

Figure S2: BNI Score After Latest Treatment Analysis………………………………………………………

Figure S3: BNI Score After Latest Follow-up Analysis……………………………………………………

Figure S4: Pain Relief Analysis……………………………………………………………………………..

Table S2: Pain Relief Meta-Regression Analysis……………………………………………………………

Figure S5: Pain Recurrence Analysis………………………………………………………………………..

Table S3: Pain Recurrence Meta-Regression Analysis……………………………………………………...

Figure S6: Facial Hypoesthesia Analysis……………………………………………………………………

Figure S7: Funnel Plots……………………………………………………………………………………...

Table S1. Search Strategy

Database	Query	Results
PubMed	(("repeat" OR "recurrent"OR "salvage") AND (radiosurgery OR "stereotactic radiosurgery" OR "Gamma Knife" OR GKRS OR "gamma-knife" AND trigeminal)	237
Scopus		142
MEDLINE		996
Web of Science	(("repeat" OR "recurrent"OR "salvage") AND ("Gamma Knife" OR GKRS OR "gamma-knife" AND trigeminal)	1,746

Figure S1. BNI Score After Prior Treatment Analysis. A) Forest plot. B) Leave-one-out analysis.

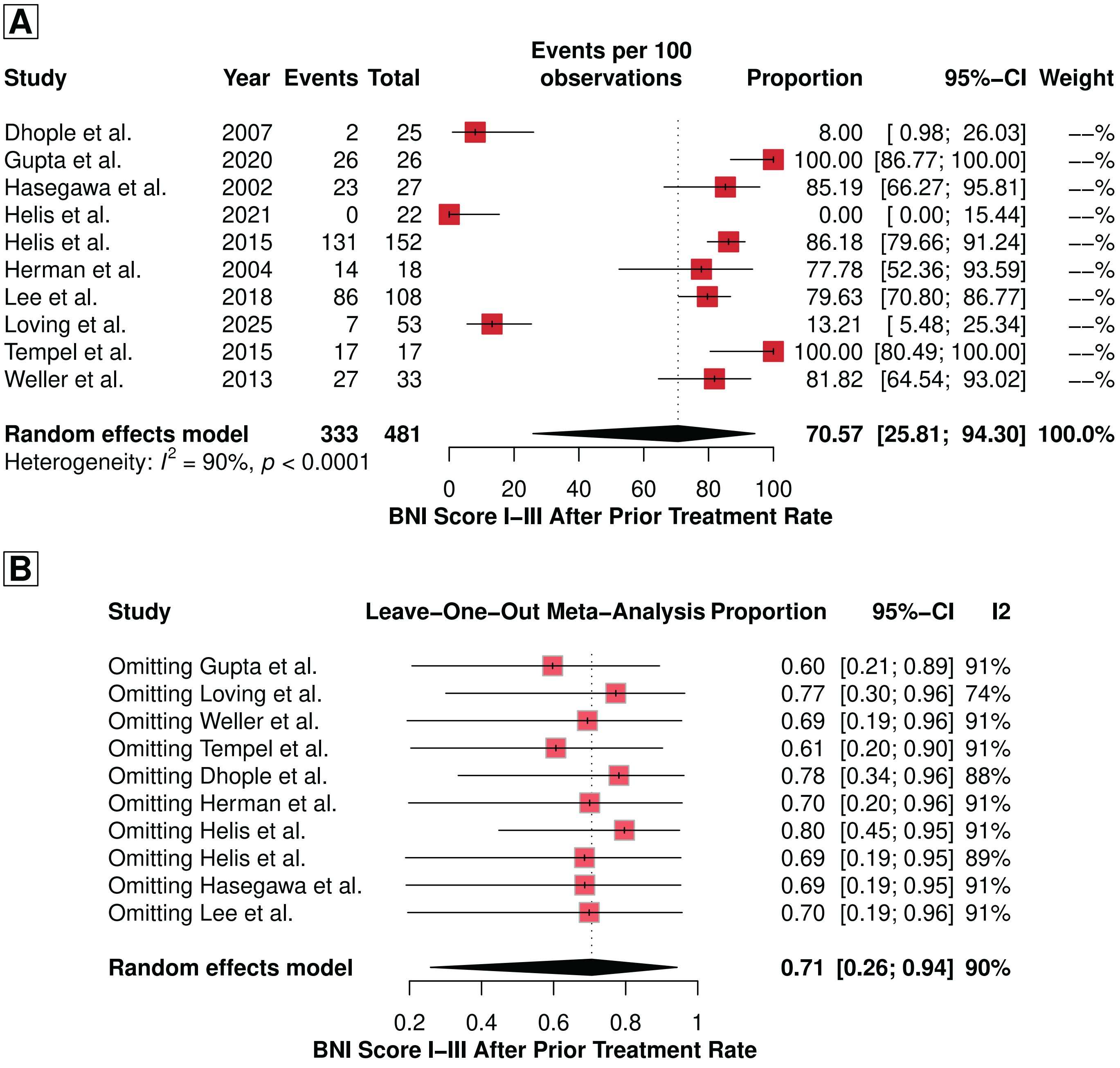

Figure S2. BNI Score After Latest Treatment Analysis. A) Forest plot. B) Leave-one-out analysis.

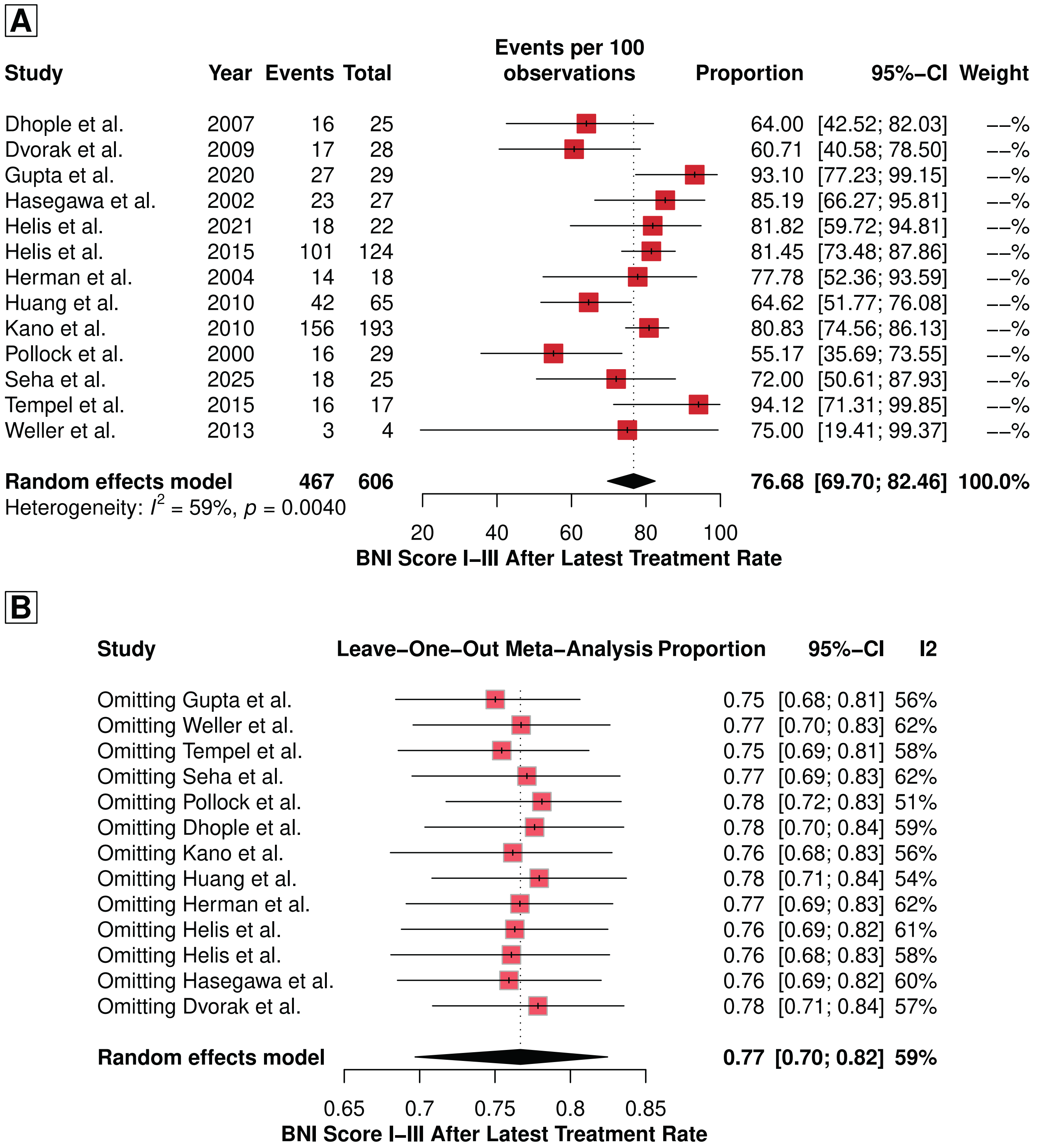

Figure S3. BNI Score After Latest Follow-up Analysis. A) Forest plot. B) Leave-one-out analysis.

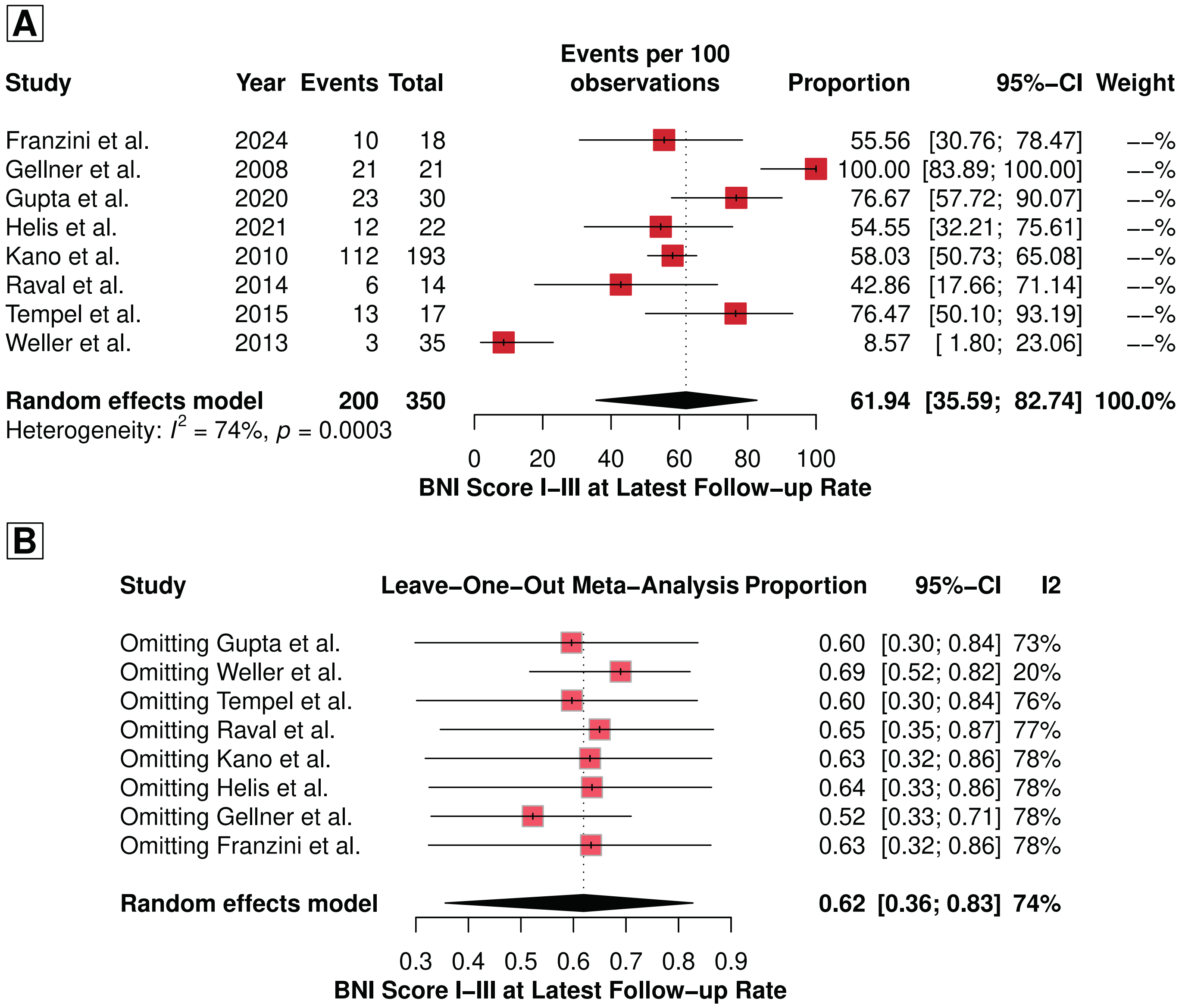

Figure S4. Pain Relief Analysis. A) Forest plot. B) Leave-one-out analysis.

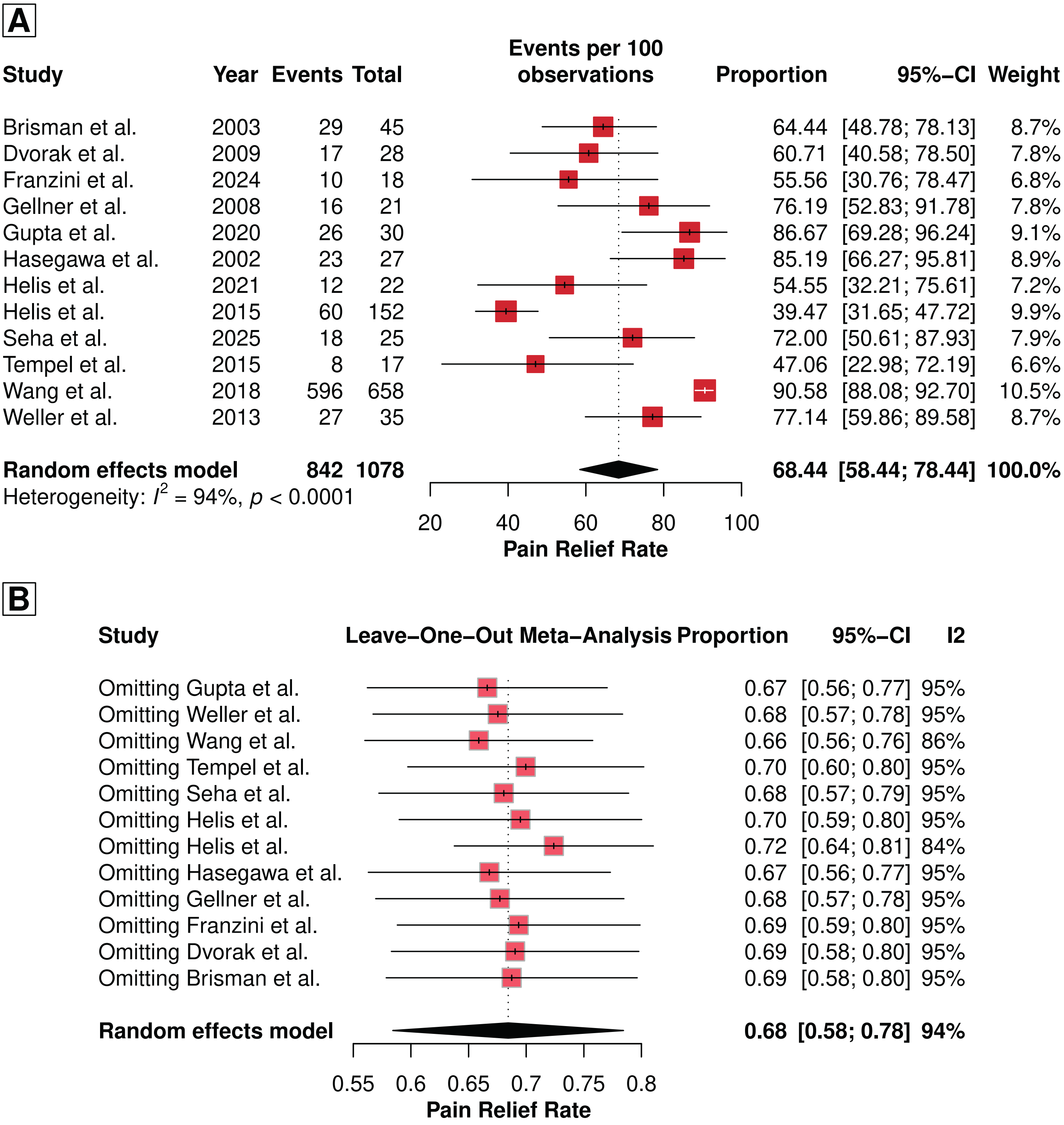

Table S2. Pain Relief Meta-Regression Analysis.

Variable	N of studies	Effect Estimate	p-value	I^2	Test for Residual Heterogeneity
Intercept	K= 6	1.2545	0.1757	91.77%	P < .0001
AgeFS		-0.0086	0.5682		
Intercept	K= 8	0.4518	0.5581	80.61%	P < .0001
AgeLS		0.0022	0.8433		
Intercept	K= 6	0.6416	<.0001	55.61%	P < .0001
PainTime		0.0012	0.3593		
Intercept	K= 8	0.5256	0.0019	81.39%	P < .0001
LastGKTime		0.0047	0.4093		
Intercept	K= 10	0.7189	0.0085	80.31%	P < .0001
Dose		-0.0011	0.7857		

Figure S5. Pain Recurrence Analysis. A) Forest plot. B) Leave-one-out analysis.

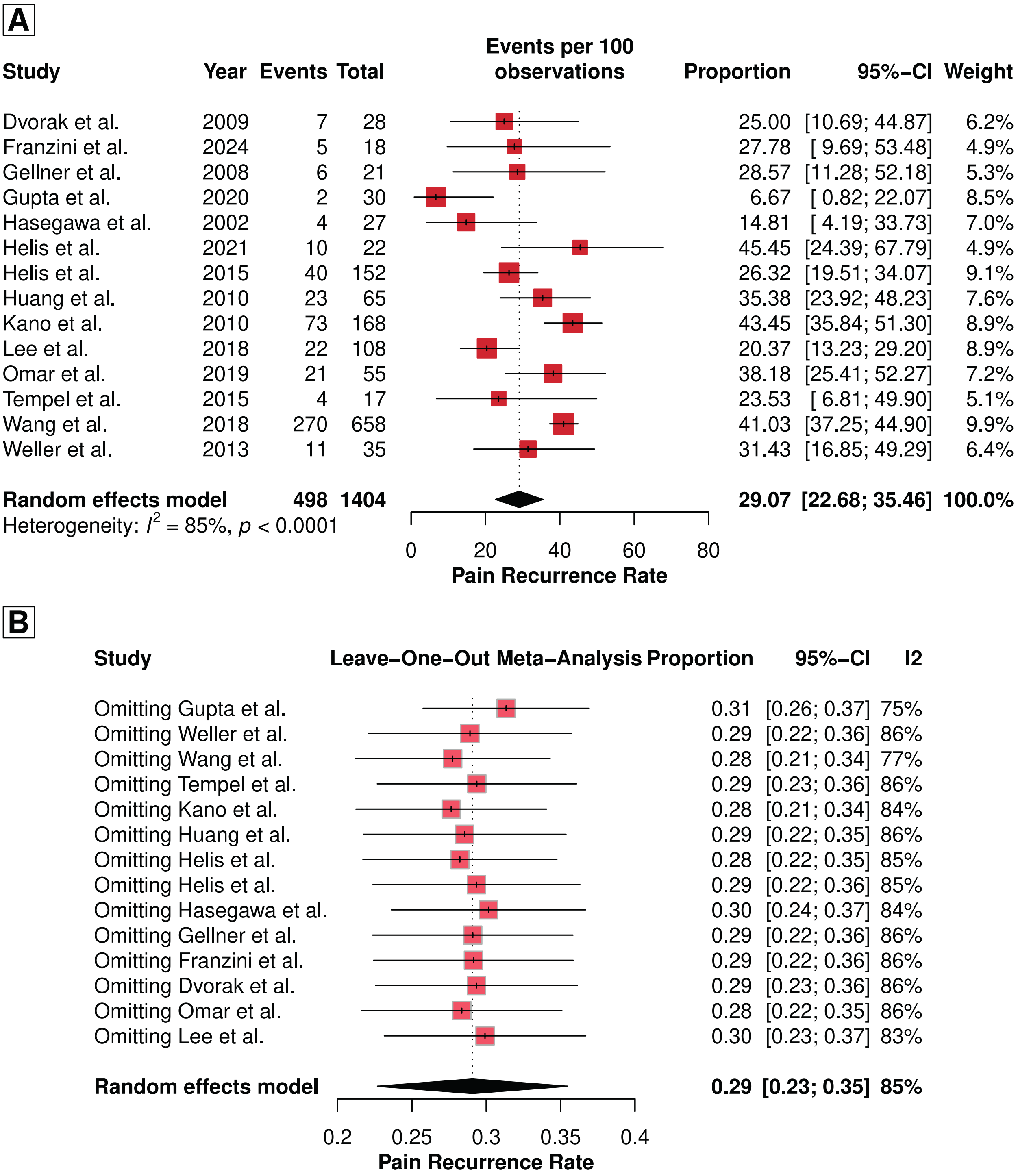

Table S3. Pain Recurrence Meta-Regression Analysis.

Variable	N of studies	Effect Estimate	p-value	I^2	Test for Residual Heterogeneity
Intercept	K= 9	0.9444	0.0491	82.93%	P < .0001
AgeFS		-0.0109	0.1632		
Intercept	K= 7	0.6611	0.2484	73.20%	P < .0001
AgeLS		-0.0057	0.4857		
Intercept	K= 8	0.3110	0.0192	84.40%	P < .0001
PainTime		-0.0004	0.7474		
Intercept	K= 10	0.1714	0.1255	75.12%	P < .0001
LastGKTime		0.0035	0.2653		
Intercept	K= 10	0.2766	0.2513	78.48%	P < .0001
Dose		-0.0000	0.9889		

Figure S6. Face Hypoesthesia Analysis. A) Forest plot. B) Leave-one-out analysis.

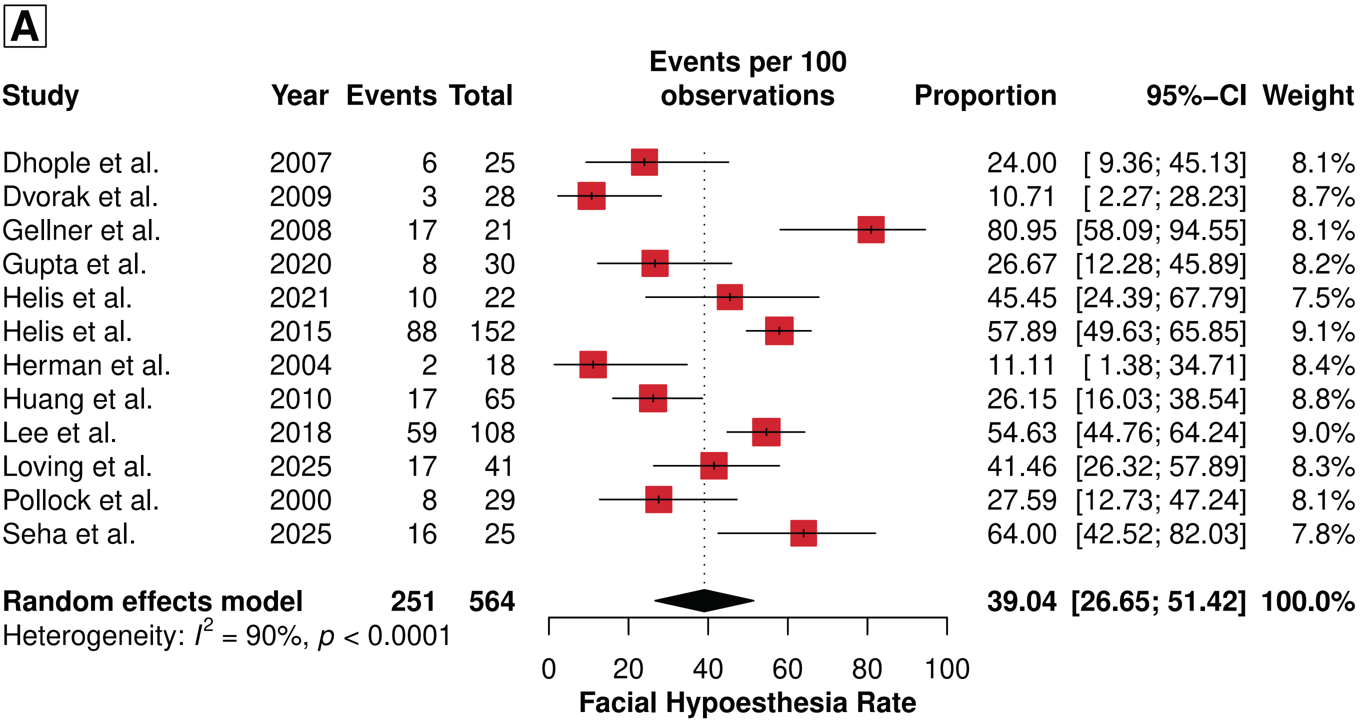

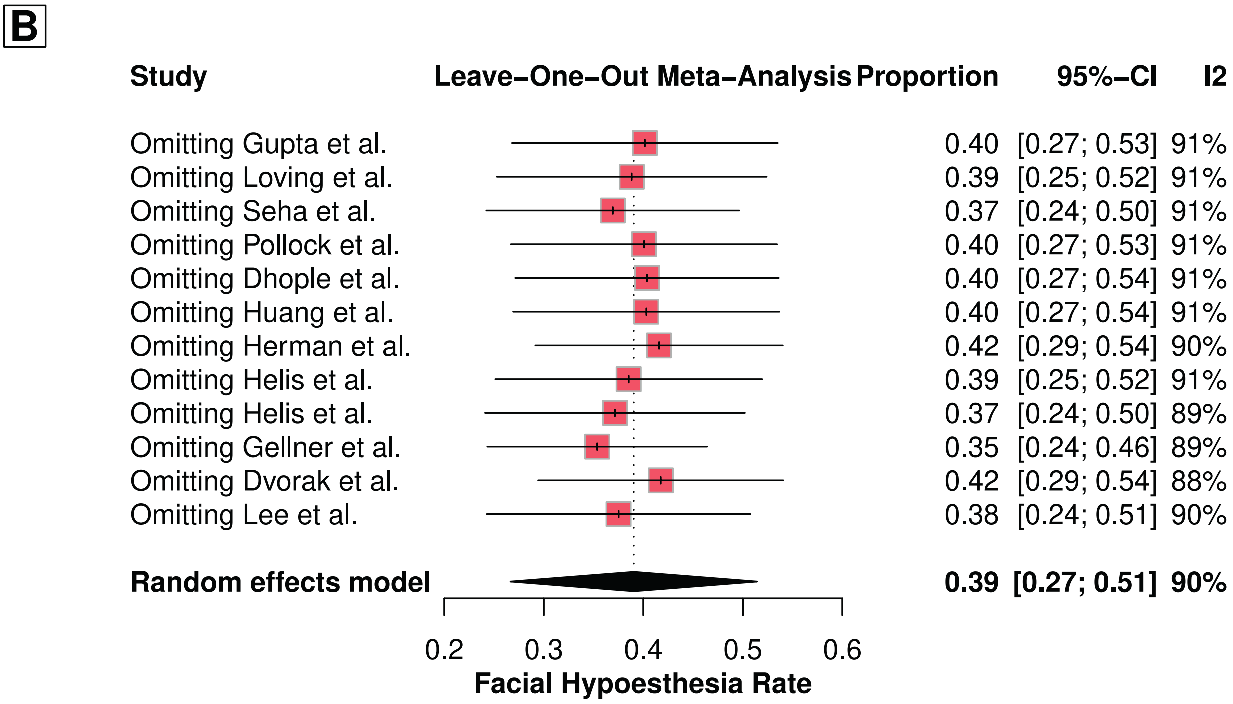

Figure S7. Funnel Plots. A) BNI Score After Prior Treatment. B) BNI Score After Latest Treatment. C) Pain Relief. D) Pain Recurrence. E) Facial Hypoesthesia.

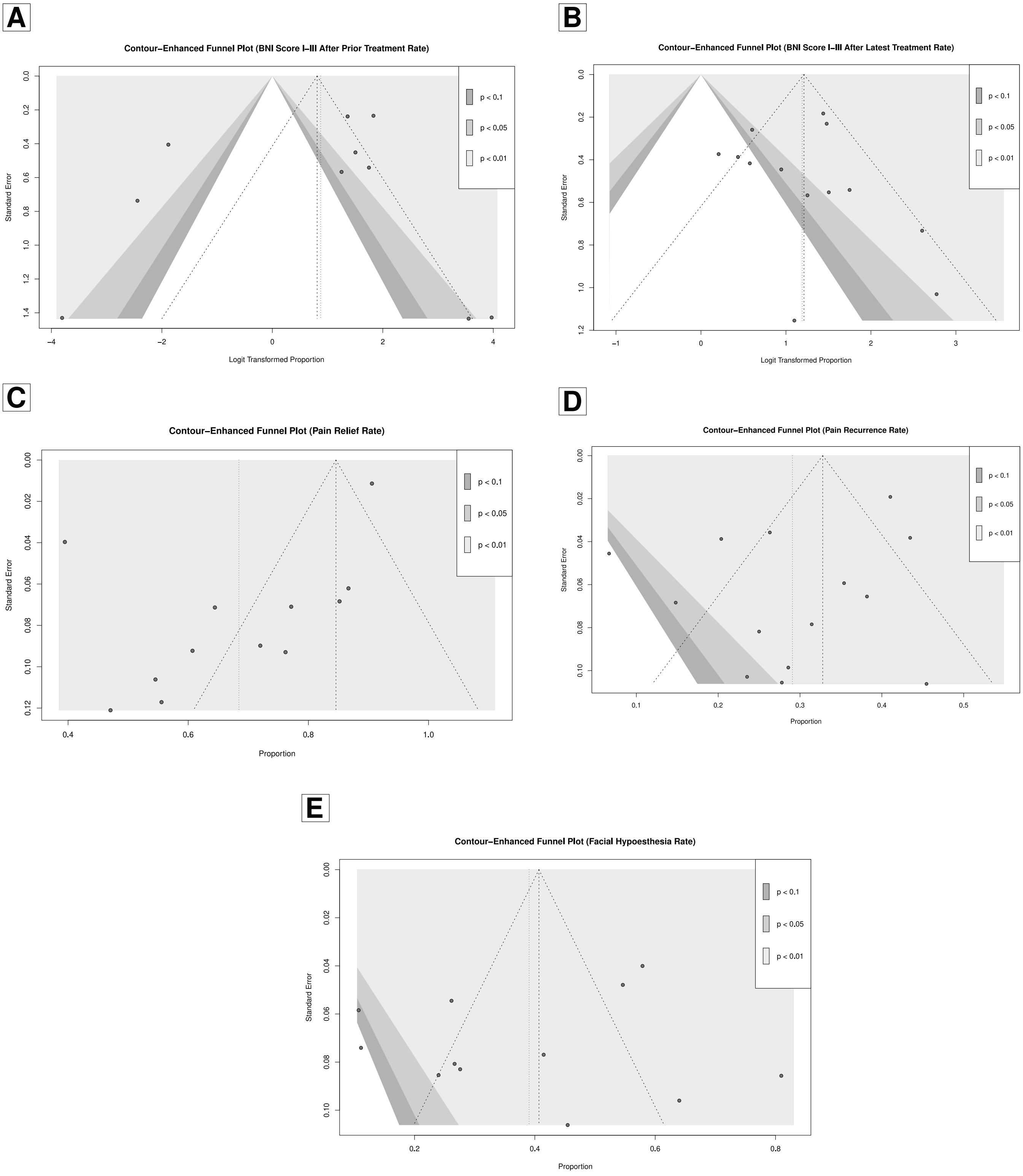

Supplement: Supplementary file 1 — Supplementary Material 1 (PDF 1.05 MB) [file 701_2026_6891_MOESM1_ESM.pdf]
